# Outcomes of renal transplantation in patients with AL amyloidosis: an international collaboration through The International Kidney and Monoclonal Gammopathy Research Group

**DOI:** 10.1038/s41408-022-00714-5

**Published:** 2022-08-18

**Authors:** Andrea Havasi, Cihan Heybeli, Nelson Leung, Avital Angel-Korman, Vaishali Sanchorawala, Oliver Cohen, Ashutosh Wechalekar, Frank Bridoux, Insara Jaffer, Victoria Gutgarts, Hani Hassoun, Maya Levinson, Cara Rosenbaum, Paolo Milani, Giovanni Palladini, Giampaolo Merlini, Ute Hegenbart, Stefan Schönland, Kaya Veelken, Alexander Pogrebinsky, Gheorghe Doros, Heather Landau

**Affiliations:** 1 Amyloidosis Center, Boston University School of Medicine, Boston, MA USA; 2 Mayo Clinic, Rochester, MN USA; 3 National Amyloidosis Centre, University College London, London, UK; 4 Department of Nephrology, CIC INSERM 1402, CHU Poitiers, Poitiers, France; 5 Centre national de référence Amylose AL & autres maladies par dépôts d’immunoglobulines monoclonales, CHU Poitiers, Poitiers, France; 6 Centre National de la Recherche Scientifique UMR CNRS 7276/INSERM U1262, Université de Limoges, Limoges, France; 7 Memorial Sloan Kettering Cancer Center, New York, NY USA; 8 Weill Cornell Medicine, New York, NY USA; 9 Amyloidosis Research and Treatment Center, Foundation “Istituto di Ricovero e Cura a Carattere Scientifico (IRCCS) Policlinico San Matteo” and Department of Molecular Medicine, University of Pavia, Pavia, Italy; 10 Amyloidosis Center, Heidelberg University Hospital, Heidelberg, Germany; 11 Department of biostatistics, Boston University School of Public Health, Boston, MA USA

**Keywords:** Haematological cancer, Medical research

## Abstract

Effective systemic therapies suppress toxic light chain production leading to an increased proportion of patients with light chain (AL) amyloidosis who survive longer albeit with end-stage renal disease. There is a critical need to identify patients in this population who benefit from renal transplantation. This multicenter, observational study from five countries includes 237 patients with AL amyloidosis who underwent renal transplantation between 1987 and 2020. With a median follow-up of 8.5 years, the median overall survival from renal transplantation was 8.6 years and was significantly longer in patients with complete and very good partial hematologic responses (CR + VGPR) compared to less than VGPR (9 versus 6.8 years; HR: 1.5, *P* = 0.04 [95% CI: 1–2.1]) at renal transplantation. Median graft survival was 7.8 years and was better in the CR + VGPR group (8.3 vs 5.7 years, HR: 1.4, *P* = 0.05 [95% CI: 1–2]). The frequency and time to amyloid recurrence in the graft was also lower (16% vs 37%, *p* = 0.01) and longer (median time not achieved vs 10 years, *p* = 0.001) in the CR + VGPR group. Comparing CR vs. VGPR there was no difference in overall or graft survival. Although 69 patients (29%) experienced hematologic relapse, treatment effectively prevented graft loss in the majority (87%). Renal transplantation in selected AL amyloidosis patients is associated with extended overall and renal graft survival. Patients with hematologic CR or VGPR have the most favorable outcomes, and these patients should be considered for renal transplantation.

## Introduction

Light chain (AL) amyloidosis is a result of extracellular deposition of pathologic, insoluble fibrils that are formed from abnormal light chains (LCs) or LC fragments. It is a systemic disease that affects various organs and is associated with an underlying clonal B-cell disorder (most commonly plasmacytic) [1, 2]. The accumulation of amyloid fibrils disrupting tissue structure and direct toxicity from circulating precursors eventually leads to organ failure and death [2–4]. Approximately 57–80% of AL amyloidosis patients have renal involvement at presentation, and up to ~40% of these patients require renal replacement therapy during the course of their disease [5–8]. A renal staging system for the prediction of ESRD using universally available measurements (i.e., proteinuria and eGFR) was validated in patients with AL amyloidosis and being used in clinical practice [7].

Until the mid-1990s, the treatment of AL amyloidosis was ineffective, resulting in a median overall survival (OS) of only 12–18 months [9, 10]. Over the last two decades, newer therapeutic regimens directed towards the toxic plasma cell clone have become available, including high dose melphalan with autologous stem cell transplantation (HDM/SCT), proteasome inhibitors (bortezomib, ixazomib), immunomodulatory drugs (pomalidomide, lenalidomide, thalidomide) and monoclonal antibodies (daratumumab) [2, 8, 11, 12]. Due to these breakthroughs in treatment, there is now a higher frequency of complete hematologic response (CR) or very good partial response (VGPR), which has translated into improved organ response and OS [7, 8, 12–14].

Longer survival led to increased number of patients who progress to end stage renal disease (ESRD). Referral for renal transplantation in these patients has been hampered due to the initial experience of early graft loss and shorter OS from recurrence of amyloidosis in graft and/or extra-renal organs compared to patients who received renal allografts for non-amyloid indications [15, 16]. However, because of prolonged disease-free survival in the setting of effective plasma cell-directed therapies, renal transplantation has become a more realistic option.

A few amyloidosis referral centers have published their individual experience with kidney transplantation in AL amyloidosis patients, but data from larger, combined studies are lacking. A major challenge faced by transplant centers is the lack of uniform criteria to determine which patients would be suitable candidates for renal transplantation. This international collaboration set out to expand our understanding of the role of renal transplantation in AL amyloidosis and define eligibility criteria based on our findings. We analyzed the demographic, clinical, and biochemical data collected on 237 patients followed at various centers in the United States, England, France, Italy, and Germany over the past 30 years who underwent renal transplantation for AL amyloidosis.

## Methods

A total of 237 patients with ESRD from AL amyloidosis who underwent renal transplantation at various centers were included (see centers under the author’s affiliations). Patients were followed between November 1987 and September 2020. From smaller previously published series 175 patients were included [17–21]. Data were collected both prospectively and retrospectively and analyzed retrospectively. The study was approved by the Institutional Review Board in accordance with the Declaration of Helsinki and the Health Insurance Portability and Accountability Act guidelines. Only de-identified data were shared. All patients had renal involvement as evidenced by biopsy-proven amyloid deposition in their kidney, or were showing typical signs of AL amyloidosis involving the kidney (urine protein >0.5 g/day and/or renal insufficiency without other causes of proteinuria) combined with tissue diagnosis at an alternate site such as abdominal fat pad [22]. Amyloid typing was done by immunohistochemistry, immunofluorescence, immunoelectron microscopy or proteomics analysis [2]. A monoclonal light chain of the same isotype needed to be detected in the deposits and in the plasma or urine (by serum or urine immunofixation or circulating free light-chain (FLC) quantitation) [23]. Patients with multiple myeloma were excluded. After 2003 hematologic response was defined as CR, VGPR, PR, or NR as described previously [22, 24, 25]. Before 2003 a hematologic response relied on serum and urine protein electrophoresis (SPEP & UPEP) and immunofixation (IFE) as well as clonal bone marrow plasmacytosis. Serum free light chain levels were prospectively followed in all patients after 2003 and free light chain assays were also done on collected (prior to 2003) frozen sera if available. Hematologic response indicates achievement of the best hematologic response which includes at least a PR although the vast majority of patients achieved a VGPR or CR. Organ involvement by amyloidosis was determined according to the international consensus criteria [22]. Glomerular filtration rate (GFR) was estimated using the four variable Modification of Diet in Renal Disease equation for patients who were <70 years of age and CKD-EPI Creatinine Equation for patients older than 70 years [26]. Histological findings were noted from biopsy reports. Allografts were biopsied as per protocol or as indicated by a decline in renal function or an increase in proteinuria. ESRD was defined as initiation of dialysis or renal transplantation. Recurrence of amyloidosis in the graft was diagnosed by a kidney biopsy or determined by clinical features of new proteinuria with or without increase in creatinine in the setting of hematologic relapse. Forty-nine patients (20%) had amyloid recurrence in the graft diagnosed.

### Statistics

Values are presented as median with range unless specified. We described study sample characteristics via counts and percentages for categorical variables. Differences between groups were evaluated using Cox regression analysis to determine the variables affecting overall and graft survival. Survival and recurrence rates were calculated using Kaplan–Meier method. The graft survival endpoint was defined by the need for permanent dialysis, second renal transplantation, or death. Sensitivity analyses were performed for the time to recurrence analysis using all-cause death as a competing risk [27]. Gray’s test was used to assess hypotheses of equality of cause-specific cumulative incidence functions between two groups. In addition, for the amyloid recurrence endpoint the estimates for the cumulative incidence functions (CIP) were also performed. Analysis was performed using SAS 9.3 software (SAS Institute INC., Cary, NC, USA) and *p* value < 0.05 was defined as statistically significant.

## Results

### Clinical features

Baseline patient characteristics are summarized in Table 1. The median age at diagnosis was 55 years (range 26–74). Sixty percent of the patients were male and 96% of them were white. Sixty-six percent of the patients had lambda light chain amyloidosis. Only 3.8% had diabetes and 32% had high blood pressure. The majority of the patients (69%) had multiorgan involvement with 41% having cardiac, 23% liver, 16% GI, 15% peripheral nervous system, and 11% autonomic nervous system involvement. Only one patient had advanced cardiac involvement (Mayo stage 3) at diagnosis, and most had Mayo stage 1. Five patients also underwent heart transplantation, and 2 patients had combined liver-kidney transplantation.

**Table 1:** Baseline patient characteristics according to hematologic response status at the time of kidney transplantation.

Characteristic	Overall (*N* = 237)	TN (*N* = 17)	CR (*N* = 145)	VGPR (*N* = 40)	PR (*N* = 24)	NR (*N* = 11)
Age at diagnosis (years; median)	55 (26–74;)	60 (34–74;)	54 (27–70)	54 (30–74)	53 (37–70)	55 (41–64)
Male	156 (65.8%)	12 (70.6%)	91 (62.8%)	27 (67.5%)	19 (79.2%)	7 (63.6%)
Race: white	228 (96%)	17 (100%)	140 (96%)	38 (95%)	23 (95%)	10 (100%)
FLC type						
Kappa	80 (34%)	6 (35%)	50 (35%)	10 (25%)	10 (42%)	4 (36%)
Lambda	157 (66%)	11 (65%)	95 (65%)	30 (75%)	14 (59%)	7 (64%)
Creatinine at diagnosis (µmol/L)	176 (35–1414)	190 (62–539)	197 (35–1414)	108 (35–1061)	135 (62–875)	163 (53–371)
eGFR MDRD at diagnosis	35.5 (2–139)	27 (9–97)	30 (2–139)	59 (4–135)	61.5 (6–127)	42 (16–124)
Proteinuria (grams/24 hr)	7 (0–41.8)	3.7 (0.2–26)	7.235 (0–41.8)	5.6 (0–17.6)	7.99 (0–23.2)	7.4 (0–15.3)
Co-morbidities						
DM	9 (3.8%)	0 (0.0%)	7 (4.9%)	1 (2.6%)	1 (4.2%)	0 (0.0%)
Hypertension	76 (32.3%)	8 (47.1%)	41 (28.5%)	16 (41%)	10 (41.7%)	1 (9.1%)
Vascular disease (CAD, MI, PAD)	21 (8.9%)	2 (11.8%)	14 (9.7%)	4 (10.3%)	0 (0.0%)	1 (9.1%)
Organ involvement						
Cardiac	96 (40.5%)	5 (29.4%)	57 (39.3%)	21 (52.5%)	8 (33.3%)	5 (45.5%)
Renal	226 (95.4%)	17 (100%)	136 (93.8%)	38 (95%)	24 (100%)	11 (100%)
Hepatic	55 (23.2%)	4 (23.5%)	34 (23.4%)	8 (20%)	9 (37.5%)	0 (0.0%)
GI	38 (16%)	3 (17.6%)	20 (13.8%)	8 (20%)	5 (20.8%)	2 (18.2%)
AN	27 (11.4%)	3 (17.6%)	11 (7.6%)	6 (15%)	5 (20.8%)	2 (18.2%)
PN	36 (15.2%)	4 (23.5%)	17 (11.7%)	7 (17.5%)	6 (25%)	2 (18.2%)
Pulmonary	2 (0.8%)	0 (0.0%)	1 (0.7%)	1 (2.5%)	0 (0.0%)	0 (0.0%)
Multi organ involvement	162 (68.4%)	9 (52.9%)	95 (65.5%)	31 (77.5%)	18 (75%)	9 (81.8%)
Type of renal transplantation						
Living	131 (54%)	14 (87.5%)	82 (57.7%)	23 (60.5%)	8 (36.4%)	4 (36.4%)
Cadaveric	106 (46%)	3 (18%)	63 (43%)	17 (42%)	16 (66%)	7 (63.6%)

*CR* complete response, *VGPR* very good partial response, *PR* partial response, *NR* no response, *TN* treatment naive.

The median serum creatinine of the patients who were not on dialysis at diagnosis was 2 mg/dL (range: 0.4–20; 176 µmol/L (range: 35–1414)), eGFR was 35.5 mL/min/1.73 m^2^ (range 4–139), and proteinuria was 7 g per 24 h (range 0–41). The median time from diagnosis to end-stage renal disease was 1.6 years (range 0–11.5). Median overall survival from diagnosis was 14.3 years (range 1–32).

### Treatment outcomes at the time of kidney transplantation

**Fig. 1:**
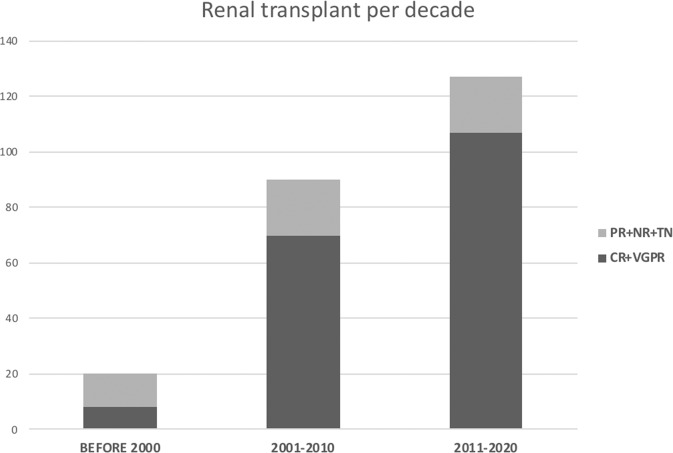
Renal transplantation per decade. Number of renal transplantations per decade according to hematologic status at the time of renal transplantation: CR + VGPR vs. PR + NR + TN (CR complete response, VGPR very good partial response, PR partial response, NR no response, TN treatment naive).

The number of patients undergoing renal transplantation has increased over the last 30 years (Fig. 1). There were 20 (8%) patients transplanted before 2000, 91 (38%) between 2000 and 2010 and 128 (53%) between 2011 and 2020. In addition, there have been an increasing number of patients who achieved CR or VGPR as a result of successful treatment (Fig. 1). Hematologic response status at the time of kidney transplantation was as follows: 145 patients with CR, 40 with VGPR, 24 with PR, 11 with NR and 19 were treatment naive (Table 1).

The majority of the patients (62%) underwent HDM/SCT. Baseline patient characteristics were not different between the patients treated with HDM/SCT or chemotherapy alone except eGFR at diagnosis which was better in the SCT group (46 vs. 34 ml/min/1.73 m^2^, *p* = 0.01). The percentage of patients who underwent HDM/SCT at the different centers varied between 40% and 100%. HDM/SCT was the first line therapy in 75 patients (31%). All five patients with combined heart and kidney transplantation were treated with HDM/SCT. From the 148 patients who underwent HDM/SCT at any time point, 31 were treated after renal transplantation either as first-line treatment (treatment-naive patients; *N* = 14) or for relapsed disease.

Seventy percent of the patients required more than one line of therapy administered before or after kidney transplantation. About 20% of the patients received 3, 14% received 4 or more rounds of treatment.

### Renal transplant outcomes

Preoperative transplant assessments and postoperative immunosuppression regimens were according to local protocol. In addition to the standard selection criteria for renal transplantation [28], most of the patients who were transplanted after 1999 required sufficient suppression of the underlying plasma cell dyscrasia to prevent the progression of amyloid organ involvement.

The median age at renal transplantation was 60.8 years (range 30.6–79).

Slightly more patients had living renal transplantation than cadaveric donation (54% vs. 46%). In the United States, living donation was more common than in the European countries (% living donation: 73% vs 28%; USA vs Europe). There were 33 patients (13.8%) with preemptive renal transplants. Delayed graft function was observed in 26 patients (11%). One patient had primary graft failure due to surgical complications. The vast majority of the patients were on standard maintenance immunosuppressive regimens including prednisone, tacrolimus or cyclosporine and mycophenolate mofetil. Only six patients were on a steroid-free protocol. Azathioprine was part of the regimen in six patients and sirolimus was administered in three patients. Induction therapy included thymoglobulin or basiliximab in most of the patients.

There were 10 patients who underwent two kidney transplantations and one patient had three renal transplants. The median age at the second renal transplantation was 57 years (range 46–70). The outcomes from the second and third renal transplantations are not included in the analysis.

The median time from diagnosis to renal transplantation was 3.8 years (range: −5.7–12.8), from ESRD to renal transplantation was 2 years (range 0–11.7) and from hematologic response to kidney transplantation was 2.2 years (range 0.4–10.4).

During the median follow-up of 11.7 years from diagnosis (range: 0.8–32) and 8.5 years from renal transplantation (range: 0–29), 90 patients (38%) died at the median age of 64.6 years (range: 36–81). The cause of death was reported to be directly related to amyloidosis in 31 patients. Overall survival from diagnosis was 14.4 years (95% CI: 12.8–16.3) and from renal transplantation 8.6 years (95% CI: 6.9–9.7). One-, three- and five-year OS from renal transplantation was 95%, 83% and 74%, respectively (Table 2).

**Table 2:** Comparison of major renal transplant outcomes among various patient groups.

		US [31, 32] (cadaveric /living) (All etiologies)	Europe [31, 32] (cadaveric /living) (All etiologies)	Our cohort (AL amyloidosis*)*	USRDS 2017 [33] (All types of amyloidosis)	>65 yrs US (cadaveric/ living) [31, 32] (All etiologies)	DM US (cadaveric /living) [31, 32] (All etiologies)
Number of patients				237	576		
Overall survival (years from renal Tx)	Median	N/A	N/A	8.6	5.8	N/A	N/A
	1 yr	97%/98.7%	96%/98%	95%	91%	94.2%/96%	96%/97%
	3 yrs	N/A	N/A	83%	N/A	86%/89.5%	89%/93%
	5 yrs	86%/93%	87%/94%	74%	70%	73.9%/82%	83%/87%
Graft survival (years)	Median	9.1		7.8	4.8		
	1 yr	93.4%/97.2%	90.7%/95.8%	92%	N/A	89%/94%	92%/96%
	3 yrs	N/A	N/A	79%	N/A	80%/87%	83%/88.5%
	5 yrs	72.4%/84.6%	77.8%/86.9%	69%	N/A	70%/78%	73%/81.5%

*USRDS* The United States Renal Data System, *DM* diabetes mellitus, *N/A* not applicable.

**Fig. 2:**
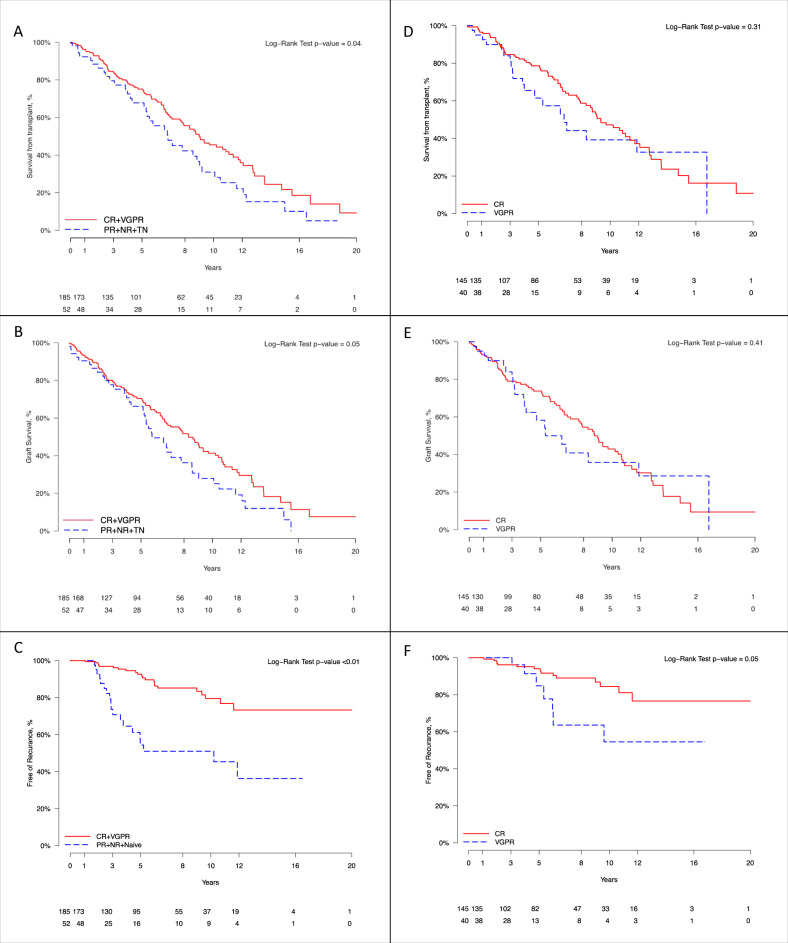
Overall survival, graft survival, and amyloid recurrence according to hematologic response status at the time of renal transplantation. **A** Overall survival from renal transplantation: CR + VGPR vs. PR + NR + TN. **B** Graft survival: CR + VGPR vs. PR + NR + TN. **C** Time from renal transplantation to recurrence of amyloid in the graft: CR + VGPR vs. PR + NR + TN. **D** Overall survival from renal transplantation: CR vs. VGPR. **E** Graft survival: CR vs VGPR. **F** Time from renal transplantation to recurrence of amyloid in the graft: CR vs. VGPR. (CR - complete response, VGPR very good partial response, PR partial response, NR no response, TN treatment naïve).

Survival outcomes were analyzed based on the patients’ degree of hematologic response to therapy at the time of renal transplantation. OS from renal transplantation was 9 years in patients with CR + VGPR and 6.8 years in patients with PR + NR + TN (HR: 1.5, *P* = 0.04 [95% CI: 1–2.1]) (Fig. 2A). When comparing CR vs. VGPR there was no difference in OS from renal transplantation (Fig. 2D; OS from renal transplantation CR vs. VGPR: 9 vs. 6.7 years, *P* = 0.3).

The median time of graft survival was 7.8 years (95% CI: 6.5–9). One-, three- and five-year graft survival rates were 92%, 79% and 69%, respectively (Table 2). Death censored graft survival was better in the CR + VGPR group vs the PR + NR + TN group (8.3 vs 5.7 years, HR: 1.4, *P* = 0.05 [95% CI: 1–2]) (Fig. 2B) and there was no difference between the CR and VGPR groups (Fig. 2E). There was no difference in renal transplant outcomes between patients who underwent HDM/SCT versus patients who were treated only with chemotherapy. Undergoing HDM/SCT before or after renal transplantation did not change OS or transplant outcomes. Similarly, there was no statistically significant difference between living versus cadaveric renal transplantation or between lambda versus kappa clonality. Cardiac involvement at baseline, the number of organs involved, acute rejection and preemptive renal transplant did not significantly affect patient or graft survival.

### Amyloid recurrence after renal transplantation

Sixty-nine patients (29%) experienced hematologic relapse requiring treatment post renal transplantation. Their graft survival was not statistically different from that of patients without relapse (7.8 vs. 11.7 years, *p* = 0.6). Median time to recurrence in the graft was 6.6 years. Graft loss was reported to be amyloid related only in six patients and successful hematologic treatment prevented graft loss in 87% of patients who had amyloid recurrence in the graft.

**Fig. 3:**
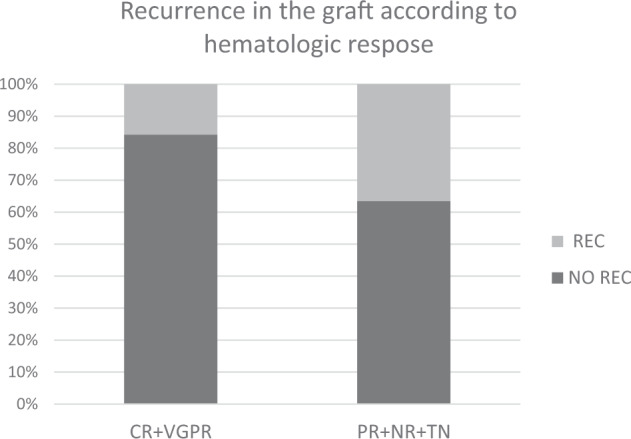
Disease recurrence in the graft after renal transplantation. AL amyloidosis recurrence in the graft after renal transplantation according to hematologic status at the time of transplantation: CR + VGPR vs. PR + NR + TN (CR complete response, VGPR very good partial response, PR partial response, NR no response, TN treatment naive).

There was a lower rate of amyloid recurrence in the graft in the CR + VGPR group compared to the PR + NR + TN group (HR: 1.6, *P* = 0.01 [95% CI: 1.1–2.5]) (Fig. 3). Using all-cause death as competing risk, the estimates for CIP for amyloid recurrence in the graft is shown in Supplemental Table). In addition, the time to recurrence in the graft was longer in the CR + VGPR vs the PR + NR + TN group and was similar between CR and VGPR patients (Fig. 2F).

### Non-amyloid related post-transplant complications

Thirty-one patients were treated for cellular rejection and seven patients had documented antibody-mediated rejection. With the exception of six patients, the majority of these rejection episodes were treated successfully without graft loss. Post-transplant lymphoproliferative disorder was documented in five patients. The incidence and types of infectious complications post renal transplantation were not available in the majority of patients.

## Discussion

New, effective systemic therapies that successfully target pathologic plasma cells and suppress toxic light chain production have led to an increase in the number of patients who survive longer albeit with ESRD. There is a lingering reluctance to offer renal transplantation to these patients due to the fear of recurrent disease and poor outcomes. Therefore, there is an urgent need to identify patients who may benefit. To address this need, we combined data from five countries under the umbrella of The International Kidney and Monoclonal Gammopathy Research Group which resulted in the largest series of patients with AL amyloidosis who underwent renal transplantation.

These data show excellent outcomes: OS from diagnosis was 14.4 years and from renal transplantation 8.6 years. The long post-diagnosis OS (compared to all AL amyloid patients) shows that these are highly selected patients with excellent response to therapeutic interventions. The median time of graft survival was also very good, 7.8 years and compared favorably to outcomes reported for patients who were transplanted for non-amyloidosis indications. This was despite the age of our patients at renal transplantation being a decade older than the average renal transplant recipient (60.8 vs. 50 years). There was no statistically significant difference between living versus cadaveric renal transplantation but this question should be revisited in a larger cohort less dependent on selection bias.

Transplant outcomes were analyzed based on the degree of hematologic response to therapy at the time of renal transplantation. Our results confirmed previous observations showing better outcomes with deeper hematologic response. Overall survival was better and amyloid recurrence rate was lower in the CR + VGPR group. Most of the patients in our cohort had achieved CR or VGPR (77%) at the time of renal transplantation suggesting a potential selection bias. Patients in the PR, NR, and TN groups were mainly from the era when therapeutic options were limited and therefore, not surprisingly, their outcomes are worse. Importantly, these cases provide insight into the natural history of AL amyloidosis after renal transplantation in untreated or undertreated patients. Regardless, our results support the preferential selection of patients for renal transplantation who achieved CR and VGPR. However, median renal graft survival was still over 5 years in patients with less favorable hematologic responses at the time of transplantation. Identifying patients in this group who are most likely to benefit from renal allograft and understanding the quality of life implications of undergoing renal transplantation compared to remaining on renal replacement therapy should be the subject of future studies. Importantly, we could not find statistically significant difference in outcomes between CR and VGPR based on our cohort. Future studies should re-address this question after inclusion of more patients specially in the VGPR group.

Although patients received various treatment modalities, the majority of them (62%) underwent HDM/SCT at some point during the study period. Outcomes were superior in patients with hematologic response regardless of the treatment regimen they received. Plasma cell-directed therapy should be administered prior to renal transplantation, although a small number of patients who were treatment naive had good outcomes after undergoing HDM/SCT following renal transplantation. This group consisted of only six patients precluding any definitive conclusions from the experience of these patients.

Notably, only six allografts failed due to recurrent amyloid although one third (*n* = 69) of the patient experienced hematologic relapse after renal transplantation and required additional hematologic treatment. This proves that newer, more effective therapies can treat AL amyloidosis successfully and prevent graft loss.

The median time from ESRD to renal transplantation was 2 years and from hematologic response was 2.2 years. This time delay resulted from the combination of the wait time on the deceased donor list, the time that required to complete the transplant evaluation (both the donor and recipient in the case of living donation), observation period after completing treatment to document the durability of response and patients’ and physicians’ preferences regarding timing of the surgery. In our cohort we had no patients who were being actively treated for amyloidosis in peri-transplant period.

Cardiac and multi-organ involvement did not influence outcomes in our patient population, likely because patients with advanced extra-renal organ involvement were generally not considered for renal transplantation. In fact, only one patient who had advanced cardiac involvement (Mayo stage 3) was included in our dataset. All the patients received standard immunosuppressive regimens according to local protocols and they didn’t seem to have more frequent rejection episodes than is commonly seen in other renal transplant populations.

Previous studies have described smaller numbers patients with AL amyloidosis who underwent kidney transplantation [17–21, 29]. The Mayo Clinic reported a median OS from kidney transplantation of 10.3 years among 60 patients transplanted between 1997 and 2018 [19]. Death-censored graft survival at one- and five-years were 98.3% and 95.8%, respectively. When divided according to hematologic response, median OS was not reached for the combined CR + VGPR group and was 3.9 years for the PR + NR group. Of their 60 patients, three had allograft failure, 19 died with a functioning graft, and 13 had amyloid recurrence in the graft. The Amyloidosis Center at Boston University also reported similar median OS of 49 patients: 15.3 years from diagnosis and 10.5 years from renal transplantation [17]. One- and five-year graft survival were 94% and 81%, respectively. Patients with CR + VGPR at renal transplantation had a better OS than patients with PR + NR (17.9 versus 9.7 years; HR 2.5, *P* = 0.03). Median time to graft loss in patients with CR + VGPR was 10.4 years and with PR + NR was 5.5 years (HR 1.8, *P* = 0.15). Both patient and graft survival were comparable to outcomes achieved in non-AL amyloidosis patients. The UK National Amyloidosis Center reported 50 AL amyloidosis patients that underwent renal transplantation in the past 15 years. Sixty percent of patients had CR at the time of renal transplantation and 15% had VGPR. Overall survival from renal transplantation was 9 years [18]. In their cohort patients who achieve CR had better survival than patients with VGPR which led to their conclusion that patients should achieve CR before consideration for renal transplantation. In a smaller cohort from Memorial Sloan Kettering Cancer Center, all treated with HDM/SCT, at the time of renal transplantation 14/16 patients had achieved a hematologic VGPR or better while the other two had hematologic progression. During a median follow-up time of 10 years, four patients experienced hematological progression and all four successfully received salvage therapy [21].

While single-center studies offer important insight about certain populations, one of the major strengths of our study is the inclusion of patients from five different countries who were not necessarily treated uniformly. The extended follow-up, 11.7 years from diagnosis and 8.5 years from renal transplantation and the number of patients included, resulting in the largest published series, are other notable strengths.

While this study represents the largest published cohort to date, we acknowledge several limitations that decrease the generalizability of our findings. The vast majority of patients included were Caucasian. This bias is mainly due to the demographics of the countries participating in the study but, under-diagnosis of AL amyloidosis in other ethnicities and the racial and ethnic disparities in access to renal transplant likely also contributed [30]. Another factor that led to heterogeneity in identifying amyloid recurrence in the graft includes protocol versus clinically indicated biopsies. Protocol biopsies might have led to earlier detection and treatment of amyloid recurrence in the graft when compared to patients whose grafts were biopsied only when it was clinically indicated. Treatment decisions regarding modalities and timing were physician dependent and might have led to bias. Eligibility for kidney transplantation was also determined locally potentially leading to variability in patient selection. Lastly, a large majority of the data was collected retrospectively.

Despite these limitations, our results clearly show that selected patients with AL amyloidosis undergoing kidney transplantation have excellent outcomes. Our consensus is that patients who achieve CR or VGPR using the current International Society of Amyloidosis criteria [25] should be considered for renal transplantation if other standard renal transplant criteria are also met. Renal transplantation prolongs survival and offers a much-needed improvement in the quality of life for these patients. However, close monitoring and carefully selected therapeutic strategies should be administered by a multidisciplinary team involving hematologists, nephrologists, and transplant surgeons with experience in the treatment of this rare disease.

## Supplementary information

Suppl Table 1

## Data Availability

The datasets generated during and/or analysed during the current study are available from the corresponding author on reasonable request.
