# Supplementary material for: Outcomes of renal transplantation in patients with AL amyloidosis: an international collaboration through The International Kidney and Monoclonal Gammopathy Research Group
Source: Blood Cancer J. 2022 Aug 18;12(8):119. doi: 10.1038/s41408-022-00714-5 (PMC9388492; doi:10.1038/s41408-022-00714-5)
Supplement: Supplementary file 1 — Suppl Table 1 [file 41408_2022_714_MOESM1_ESM.docx]

**Suppl Table 1A: Estimates for amyloid recurrence rates according to hematologic response status at the time of renal transplantation: CR+VGPR vs. PR+NR+TN (CR: complete response, VGPR: very good partial response, PR: partial response, NR: no response, TN: treatment naive)**

| **Groups** | **Time Points** | **Rates with All-Cause death as Competing risk** | **Rates with All-Cause death as censoring** |
| --- | --- | --- | --- |
| CR+VGPR | 0 | 0.00% | 0.00% |
| CR+VGPR | 1 | 0.00% | 0.00% |
| CR+VGPR | 3 | 2.85% | 3.08% |
| CR+VGPR | 5 | 6.27% | 7.40% |
| CR+VGPR | 6 | 10.88% | 13.71% |
| CR+VGPR | 8 | 11.68% | 14.84% |
| CR+VGPR | 10 | 14.53% | 20.45% |
| CR+VGPR | 12 | 17.42% | 26.69% |
| CR+VGPR | 14 | 17.42% | 26.69% |
| CR+VGPR | 16 | 17.42% | 26.69% |
| CR+VGPR | 18 | 17.42% | 26.69% |
| CR+VGPR | 20 | 17.42% | 26.69% |
| PR+NR+TR | 0 | 0.00% | 0.00% |
| PR+NR+TR | 1 | 0.00% | 0.00% |
| PR+NR+TR | 3 | 22.16% | 26.31% |
| PR+NR+TR | 5 | 36.47% | 45.61% |
| PR+NR+TR | 6 | 38.86% | 49.01% |
| PR+NR+TR | 8 | 38.86% | 49.01% |
| PR+NR+TR | 10 | 38.86% | 49.01% |
| PR+NR+TR | 12 | 45.02% | 63.74% |
| PR+NR+TR | 14 | 45.02% | 63.74% |
| PR+NR+TR | 16 | 45.02% | 63.74% |
| PR+NR+TR | 18 | 45.02% | 63.74% |
| PR+NR+TR | 20 | 45.02% | 63.74% |
| Gray Test p-value <0.001 | | | |
